# Public finance management and data availability for nutrition financing in India

**DOI:** 10.1136/bmjgh-2020-004705

**Published:** 2021-04-15

**Authors:** Avani Kapur, Ritwik Shukla

**Affiliations:** Accountability Initiative, Centre for Policy Research, New Delhi, India

**Keywords:** health systems, nutrition

## Abstract

For investments to translate into improved public service delivery, having a strong public finance management (PFM) system that lays out the rules, institutions and processes by which public funds are managed is critical. To enable a better understanding of the nutrition financial landscape, this paper seeks to determine whether the current PFM system in India allows for capturing required nutrition data. It does this by mapping the availability and comparability of data for a set of key nutrition-specific interventions through the budget cycle: from budget formulation, to execution, and finally, evaluation. The study finds significant gaps in data availability including absence of financial data by level of governance, geography and intervention components. These challenges relate to gaps in PFM design in India from weak planning processes, line-item budgeting, unavailability of time costs, inefficient fund release processes, difficulties in estimating target populations and the lack of output costing. These gaps in the PFM system and consequent data issues have several implications which may lead to strained delivery. This in turn impacts quality and the possibility of course correction. Some of these challenges can be overcome by ensuring planning processes are enforced, expanding existing data systems, making more data available in the public domain, using existing research better and using assumptions carefully to cover data gaps.

**Summary box**

- For investments to translate into improved public service delivery, having a strong public finance management (PFM) system that lays out the rules, institutions and processes by which public funds are managed is critical.
- This poses important questions on the type of financial data that is generated and captured in India to enable regular tracking of nutrition finances.
- Mapping data availability across nutrition interventions, one finds that data on costs and allocations are largely available in non-disaggregated form, but complete data on expenditures is missing and also unavailable in public domain.
- Some major challenges include weak planning processes which preclude the generation of a lot of data, line-item budgeting, the absence of methodology in determining unit costs, unavailability of time costs, inefficient fund release processes, difficulties in estimating target populations and the lack of output costing.
- These gaps in the PFM system and consequent data issues have several implications which may lead to strained delivery. Some of these challenges can, however, be overcome by ensuring planning processes are enforced, expanding data collection and making more data available in the public domain.

## Introduction

In 2012, India, along with over 190 member states of the World Health Assembly, endorsed a set of global nutrition targets related to six forms of malnutrition, namely stunting, anaemia, low birth weight, childhood overweight, wasting and exclusive breast feeding. These were further endorsed as part of the Sustainable Development Goals in 2015. To achieve these, estimates suggest that globally, an additional annual investment of US$7 billion would be required between 2015 and 2025.^1^

In India, the last few years have seen attempts by the Union government at increasing budgets for nutrition including the launch of Prime Minister's Overarching Scheme for Holistic Nourishment (POSHAN) Abhiyaan and the Pradhan Mantri Matru Vandana Yojana (PMMVY), a maternity benefit scheme, in 2017. This period also saw budget increases for other nutrition schemes such as enhanced unit costs for food supplements under the Integrated Child Development Services (ICDS). Despite these changes, until 2020, there is no estimate on the total quantum of funds allocated and spent on nutrition nor the relationship between increased spending on nutrition and improved service delivery.

For investments to translate into improved public service delivery, having a strong public finance management (PFM) system that lays out the rules, institutions and processes by which public funds are managed is critical. The system can be strengthened by tracking funds through the budget cycle from budget formulation, to execution and finally, evaluation.^2–4^ If implemented well, PFM can improve the efficiency and equity of financing thereby ensuring increased provider accountability and improved outcomes quality.

Various studies suggest that stronger PFM systems correlate with better health outcomes.^5^ Given the cross-cutting and multisectoral nature of nutrition, the relationship of PFM and nutrition remains an emerging field. This poses important questions on the type of financial data that is generated and captured in India to enable regular tracking of nutrition finances.

Using the PFM framework, this paper seeks to determine whether the current PFM system in India allows for capturing required nutrition data. It does this by mapping the availability and comparability of data for a set of key nutrition-specific interventions through the budget cycle. Given that nutrition is multisectoral, recommendations on ways to improve tracking could be applicable beyond India.

The paper has two main limitations. First, it covers only key nutrition-specific interventions defined as those that address immediate determinants of nutrition. It does not include nutrition-sensitive interventions that address underlying determinants.^6^ Second, while there are multiple sources of financing for health and nutrition, this study is limited to only government sources and PFM processes at the Union government level. In a federal country like India, health and nutrition are state subjects and states spend over 60% of public health spending.^7^ However, PFM processes vary across states. Thus, for sake of comparability the focus is on the Union government.

The paper is structured as follows. Section 1 briefly looks at the instruments for nutrition financing in India and lays out the PFM framework. This is followed by highlighting data requirements for nutrition financing through the budget cycle. Section 3 reviews data availability and highlights challenges and subsequent implications. Section 4 concludes and recommends approaches to improve tracking funds for nutrition.

## Background

In India, nutrition interventions are delivered through three Centrally Sponsored Schemes (CSSs), (which are large programmes in India, funded by both union and state governments; programmatic decisions are taken by the Union government, and implementation rests with states) namely the POSHAN Abhiyaan, ICDS and the National Health Mission (NHM) run by two key ministries—the Ministry of Women and Child Development (MWCD) and the Ministry of Health and Family Welfare (MoHFW) (table 1).

**Table 1:** Direct nutrition interventions by ministries

Intervention	Ministry	Scheme
**Counselling**		
Counselling during pregnancy	MWCD+MoHFW	POSHAN Abhiyaan, NHM, ICDS
Counselling for breast feeding (0–6 months)	MWCD+MoHFW	POSHAN Abhiyaan, NHM, ICDS
Counselling for CF and WASH	MWCD+MoHFW	POSHAN Abhiyaan, NHM, ICDS
**Food supplements**		
Food supplements for adolescent girls	MWCD	SAG
Food supplements for pregnant women	MWCD	ICDS
Food supplements for lactating women	MWCD	ICDS
Food supplements for children	MWCD	ICDS
Food supplements for malnourished children	MWCD	ICDS
**Micronutrient interventions**		
IFA for adolescent girls	MoHFW	NHM
Deworming for adolescent girls	MoHFW	NHM
IFA for pregnant women	MoHFW	NHM
Calcium for pregnant women	MoHFW	NHM
Deworming for pregnant women	MoHFW	NHM
IFA for lactating women	MoHFW	NHM
Calcium for lactating women	MoHFW	NHM
Iron supplements for children (6–60 months)	MoHFW	NHM
Deworming for children (12–60 months)	MoHFW	NHM
Vitamin A supplements for children (6–60 months)	MoHFW	NHM
**Health interventions**		
ORS and therapeutic zinc supplements for treatment of diarrhoea (2–60 months)	MoHFW	NHM
Treatment of children with Severe Acute Malnutrition at Nutrition Rehabilitation Centres	MoHFW	NHM
**Maternity benefits**		
Conditional cash transfer—JSY	MoHFW	JSY (within NHM)
Conditional cash transfer—PMMVY	MWCD	PMMVY

CF, Complementary Feeding; ICDS, Integrated Child Development Services; IFA, Iron and Folic Acid; JSY, Janani Suraksha Yojana; MoHFW, Ministry of Health and Family Welfare; MWCD, Ministry of Women and Child Development; NHM, National Health Mission; ORS, Oral Rehydration Solution; PMMVY, Pradhan Mantri Matru Vandana Yojana; SAG, Scheme for Adolescent Girls; SBM, Swachh Bharat Mission; WASH, Water, Sanitation, and Hygiene.

**Figure 1:**
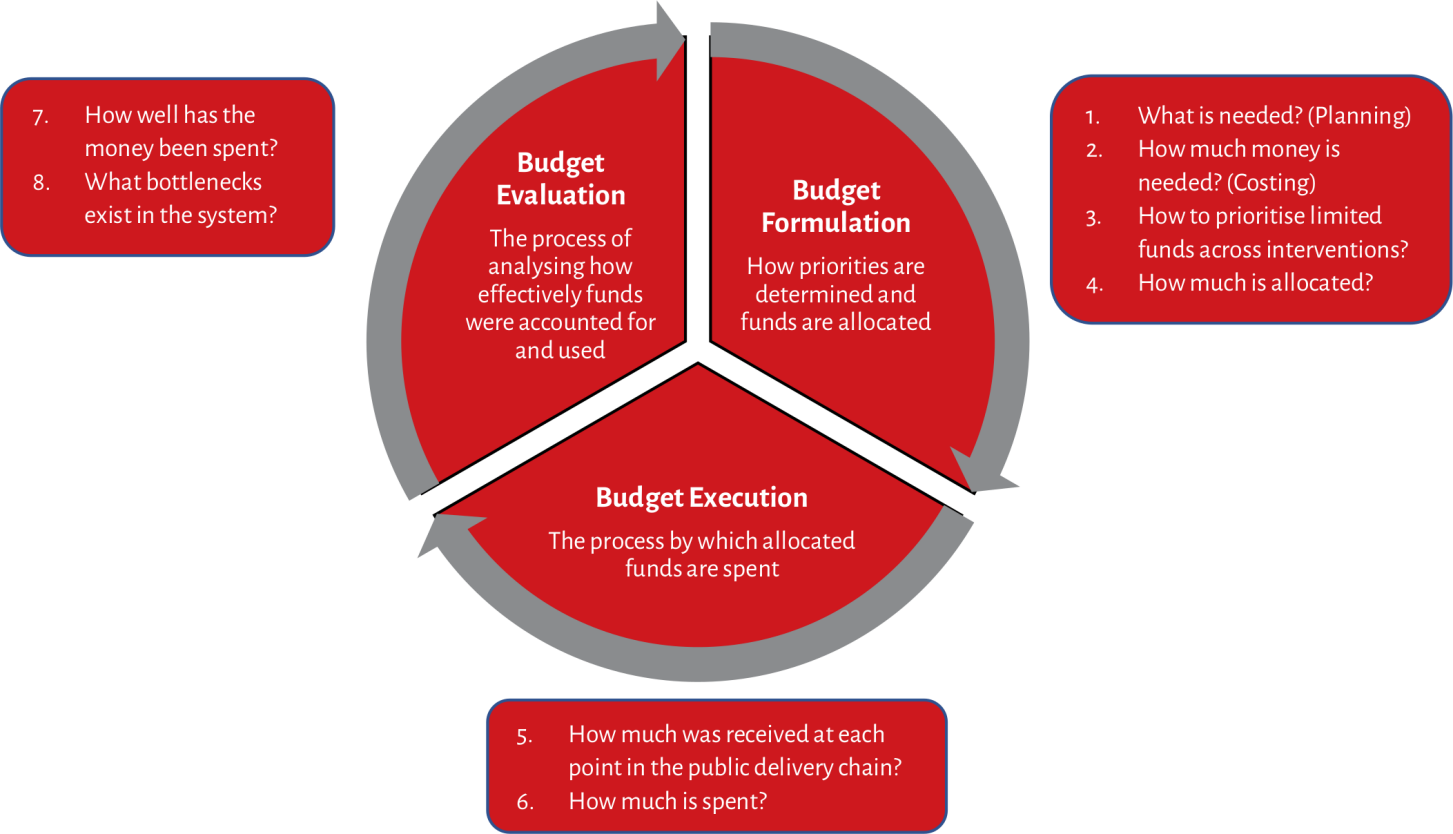
Studying finances through the nutrition budget cycle.

The PFM framework used here, based on the Public Expenditure and Financial Accountability (PEFA) framework, focusses on three stages in the budget cycle. These are: (1) Budget formulation or how public funds are planned, approved, allocated and prioritised; (2) Budget Execution or how budgets are used and (3) Budget Evaluation or how public spending is accounted for, evaluated and feeds into the next budget cycle (figure 1).^5 8 9^

For a strong and effective PFM system for nutrition the following four key steps should be possible. First, funds should be spent on the ‘right’ things, keeping in mind needs, priorities and absorptive capacity known as allocative efficiency. Second, the resources needed to deliver these interventions at scale or to 1oo% of target groups should be estimated. Third, these funds should be released on time and used effectively. Finally, implementation processes including procurement, supply chain management, and ensuring adequate human resources should be simple and enable diagnosing bottlenecks with strong feedback loops.

In each step, research methods such as costing, budget analysis, expenditure tracking and diagnostic studies can play a vital role in ensuring effective delivery of nutrition interventions. Yet, underlying each of these steps is the need for intervention-wise fiscal and related information to be clearly distinguishable in disaggregated form. The next section looks at the data requirements in each of the steps in the PFM cycle. This is then followed by a mapping of the current availability and gaps in data for nutrition financing both intervention-wise as well as through the PFM cycle.

## Approaches and data requirements

### Budget formulation

Resources are typically scarce in low/middle-income countries like India, and expenditure at scale requires prioritisation between competing needs. Government budgets quantitatively express a policy or plan and indicate priorities.

Two useful approaches for tracking PFM processes at the budget formulation stage are costing studies to determine the total requirements needed to deliver interventions and budget analyses which offer a critical examination of the different components of allocations for the upcoming year. Taken together, they provide policy-makers the necessary tools to determine future budget needs or the relative importance of different interventions.

For instance, costing studies have been used as a starting point to estimate requirements, and assess resources necessary for scale-up.^10–12^ When combined with estimated benefits of a programme, they provide insights on cost-effectiveness.^13^ Similarly, various countries have used nutrition budget analyses in myriad ways including identifying and coordinating nutrition across sectors (Tanzania, Congo, Philippines, etc) and advocating increased funding for nutrition (Senegal, Madagascar, Nepal, etc).^14^

These studies require disaggregated intervention-wise data on needs, and unit costs at the lowest level of operation—namely the village. For costing to be holistic and equitable, these unit costs should ideally also account for personnel costs, administrative data including logistic and transport costs, and costs for monitoring. Ensuring interventions are planned and budgeted for at scale, information on the target population groups is also required. Finally, to map requirements with allocations, one needs budgets in a disaggregated manner for each intervention.

### Budget execution

Budget execution refers to the process by which allocated funds are spent. Public expenditure reviews (PERs) and public expenditure tracking (PET) surveys are important diagnostic instruments to evaluate the effectiveness of public finances by analysing government expenditures over time.^9 15^ While PERs typically assess policy priorities and intended outcomes or if funds have been spent well; PETs measure the amount of funds received at each point in the public service delivery chain, from a nation’s treasury to the last mile such as a health clinic, where the funds are to be spent.

These have been used across countries such as Tanzania, Tunisia and Indonesia^16–18^ but their use for nutrition has been limited.

Conducting PERs and PETs to feed into budget execution for nutrition requires intervention-wise data on expenditures across governance levels to find gaps. Furthermore, data are required on fund-flows including the amount and timing of funds released.

### Budget evaluation

Budget evaluation refers to the process of analysing how effectively funds were accounted for and used. Implementation science for nutrition includes several methods such as process tracking and diagnostic studies to identify and address bottlenecks and develop strategies to enhance utilisation.^19^ For instance, a study found delays in fund release as a significant contributor to poor expenditures.^20^

Data requirements on evaluation include not just information on financial and regulatory compliance but details on the extent of equity in coverage, and the outputs and outcomes achieved with existing funds. Moreover, a data management system that is flexible and allows for redressal and subsequent improvements for future budget cycles is critical.

The next section looks at the status of data available, challenges with respect to data gaps through the PFM cycle and their implications.

## Mapping data availability, gaps and implications in India

As mentioned, in India, key nutrition interventions are delivered by CSSs across two main ministries, MoHFW and MWCD. Budget formulation starts with preparing implementation plans. These are meant to be a bottom-up aggregation of needs with corresponding unit costs, starting at the village and then aggregated upwards. For the NHM these are known as Programme Implementation Plans (PIPs) and include both the physical targets, unit costs where applicable, and corresponding budgets based on an overall resource envelope. Final NHM approvals are called Record of Proceedings (RoPs).^21^ Funds approved for ICDS are in the Annual PIPs with a component-wise breakdown of different activities.^22^ Once approved by the Union government, funds are allocated and funding is shared between the Union government and States in a 60:40 ratio.

Mapping data availability across nutrition interventions, one finds that while data on costs and allocations are largely available via scheme planning and budgeting documents, these are often not disaggregated. For instance, while NHM has more disaggregated data allowing for intervention-wise analysis, ICDS budget data exclude information on key interventions such as counselling, administrative data including personnel, transport and logistic costs. Data are also available only for funds allocated by the Union government leaving a substantive gap of 40% of allocations. Further, separate data by beneficiary group is unavailable making it difficult to undertake resource estimations at scale.

Data availability is also limited for expenditures. Again, this is more available for NHM where formats known as Financial Management Reports (FMRs) capture item-wise expenditures (over 1000 in some cases) for every state in a monthly/quarterly basis. In contrast, the latest comprehensive expenditure data available including both union and state shares for ICDS is for the year 2018–2019. However, even these data are not disaggregated across components, and not available below state level (table 2).

**Table 2:** Data availability across interventions

Intervention	Data availability	Source	Latest year available	Level
**Counselling Interventions**				
Counselling during pregnancy	Not available (NA) collectively for India.	NA	–	–
Counselling for breast feeding (0–6 months)				
Counselling for CF and WASH				
**Food supplement interventions**				
Food supplements for adolescent girls	Unit costs available for costs of food provision only. Only aggregates of allocations available in scheme documents. Some aggregates of expenditure available but not publicly.	APIPs for ICDS, budget for SAG.^22 36^ Lok Sabha Questions (LSQ) (Parliament questions, posed by members of the parliament, to members of the cabinet)/Right to Information (RTI) queries (Similar to the Freedom of Information Acts in the USA.)	2020–2021 for allocations 2018–2019 for expenditures	State
Food supplements for pregnant women				
Food supplements for lactating women				
Food supplements for children				
Food supplements for malnourished children				
**Micronutrient and deworming interventions**				
IFA for adolescent girls	Unit costs and disaggregated allocations available in scheme documents. Disaggregated expenditures captured but not publicly available.	National Health Mission (ROPs 2020–21).^21^ NHM Financial Management Report (FMR).	2020–2021 for allocations 2020–2021 (till September) for expenditures (via RTI)	State
Deworming for adolescent girls				
IFA for pregnant women				
Calcium for pregnant women				
Deworming for pregnant women				
IFA for lactating women				
Calcium for lactating women				
Iron supplements for children (6–60 months)				
Deworming for children (12–60 months)				
Vitamin A supplements for children (6–60 months)				
**Health interventions**				
ORS and therapeutic zinc supplements for treatment of diarrhoea (2–60 months)	Unit costs and disaggregated allocations available in scheme documents. Disaggregated expenditures captured but not publicly available.	Costs for SAM treatment from Operational Guidelines on Facility-Based Management of Children with Severe Acute Malnutrition.^38^ Allocations from NHM (ROPs 2020–21).^21^ NHM FMR	2020–2021 for allocations 2020–2021 (till September) for expenditures (via RTI)	State
Treatment of severe acute malnutrition children at nutrition rehabilitation centres				State
**Maternity benefits**				
Conditional cash transfer—JSY	Unit costs and disaggregated allocations available in scheme documents. Disaggregated expenditures captured but not publicly available.	NHM (ROPs 2020–21).^21^ NHM FMR	2020–2021 for allocations 2020–2021 (till September) for expenditures (via RTI)	State
Conditional cash transfer—PMMVY	Allocations available at national level, as well as some unit costs. Expenditures available but not in disaggregated form.	PMMVY Union budget, 2020.^39^ LSQs/RTIs	2021–2022 for allocations Expenditure till 2019–2020 available	National for allocations State for expenditures

CF, complementary feeding; ICDS, Integrated Child Development Services; IFA, Iron and Folic Acid; JSY, Janani Suraksha Yojana; ORS, Oral Rehydration Solution; PMMVY, Pradhan Mantri Matru Vandana Yojana; ROP, record of proceeding; RTI, Right to Information; SAG, Scheme for Adolescent Girls; WASH, water, sanitation and hygiene.

These gaps highlight critical inter-related challenges in PFM processes in India which have implications for tracking nutrition finances. These are discussed below:

### Formulation

#### Weak planning and needs assessments

Scheme guidelines call for bottom-up planning, but studies have found that planning tends to be top-down resulting in a disconnect between allocations and needs on the ground.^23^ Moreover, this centralisation also exists for state plans with not all funds proposed by states are approved. For instance, in 2019–2020, 78% of combined state proposals for NHM were approved (including supplementary budgets accessed till 10 January 2020).^24^ Consequently, most unit costs follow a top-down approach, which miss local variations. For example, unit costs for food supplements are the same across India, without addressing differences across biomes in food availability, and needs of target groups.

#### Line-item budgeting

India follows input based, line-item budgeting with each department and ministry planning and budgeting systems for schemes independently. For nutrition, given its cross-cutting nature, data for interventions are spread across ministries and collating information becomes difficult. For example, counselling is split between MoHFW and MWCD and here too there is no specific budget line-item for counselling under MWCD and only certain costs such community-based events, *Jan Andolan* (People’s campaigns) are listed.

Even within a specific department/ministry, data are often spread across multiple line items. For instance, human resources under NHM are spread across several budget heads such as human resources, procurement, drug warehousing.

#### No unit cost methodology

Most government guidelines do not specify the methodology used to determine unit costs. Therefore, it is unclear how these were calculated. Furthermore, these are not updated regularly—unit costs for food supplements have remained fixed since 2017, though guidelines mention inflation-based cost indexation (This was confirmed via an RTI filed by the authors. The unit costs of food supplements per person were the same in 2017 and January 2021).^25^

#### Budgeting time costs appropriately

Ideally, resources used to provide health interventions should include costs not accounted for in a budget. These include resources which were donated, volunteered, time costs or the unpaid time spent by functionaries.^26 27^ While rarely accounted for in the budget process, these should be considered while deciding salary and honorarium amounts within the budget. Remuneration is a source of worker motivation. If functionaries are paid for less time than they work, they can feel overburdened thereby leading to dissatisfaction which could further affect performance.^28^

### Execution

#### Inefficient fund releases

There are several delays through the fund approval and fund flow process. A study found that at the state level alone, a paper file for fund releases had to pass across a minimum of 32 desks.^20^ Fund release delays and line-item budgeting further affect coverage and equity. It is likelier that routine expenditure such as salaries and administrative costs are prioritised as opposed to other activities that may also be crucial but more complex.

#### Limited expenditure information

Schemes require Utilisation Certificates (UCs) to be submitted for expenditures incurred. These UCs, however, do not capture the quality of expenditure and are often not in machine-readable forms, limiting their availability and usage. Further, expenditure information is rarely available publicly thereby limiting accountability.

### Evaluation

#### Difficulties in estimating target population

Most interventions are rolled out selectively for specific population groups, specific geographies and different levels of coverage. India has three main sources for population data—Census 2011, Health Management Information System (HMIS) and a 2019 report of the technical group on population projections.^29–31^ Census 2011 is outdated, the report has limitations including underestimating population for latter years (this is due to the use of several assumptions regarding fertility, mortality, and migration which may not hold in future years, as acknowledged by the authors of the report as well) and lack of age-wise estimates across states and years, and HMIS suffers from quality issues.^32–34^

#### Lack of output costing

Unit costs typically refer to inputs rather than costs involved in all necessary processes. This can hamper outcomes. For instance, ineffective processes in delivering food and not accounting for them restricts achieving outcomes even when budgets are adequate.

#### Implications

These gaps in the PFM system and consequent data issues have several implications. Without data on needs, requirements may be over or underestimated. Not accounting for time and administrative costs can strain delivery, impacting quality. A lack of data on the amount and timing of releases has an impact on spending and makes it difficult to track priorities and expenditure gaps. A lack of data on processes makes it difficult to analyse how to improve system functioning and efficiency. These in turn affect the possibility of course correction (table 3).

**Table 3:** Data gaps and consequent implications

	Gaps	Implications
**Budget formulation**		
What is needed? (Planning)	Plans are often not made in a disaggregated manner, and therefore data on village level needs is not available. There are no adequate needs assessments.	Limited information on local requirements impacts prioritisation and equity of financing.
How much money is needed? (Costing)	Budgeting is undertaken in silos across departments not allowing for comprehensive assessment of costs. For some interventions like counselling, no disaggregated usable data are available for India. Lack of disaggregated data on personnel and time costs which reflect the amount of time they need to spend on each task, administrative costs cover processing paperwork, ensuring funds reach on time, and so on logistics and transport costs, and monitoring and testing costs.	Absence of information increases reliance on assumptions which can lead to an over or underestimation of costs required. Not accounting for personnel and time costs can lead to overworking existing workers resulting in inefficiencies in implementation. Similarly, not accounting for administrative costs can cause delays which affects programme timeliness and quality.
How to prioritise limited funds across interventions?	Globally, it is evident that counselling and micronutrient interventions are more cost-effective yet such cost-effectiveness studies are limited in India.	Interventions which have greater effectiveness should be prioritised but there is a lack of India-specific evidence. Failing that, the number of lives saved, or the number of days of diseases averted may be lower than possible.
How much is allocated?	Data not always publicly available disaggregated by components as with food supplements, nor is disaggregated by levels of government. State specific allocations are usually excluded, which may be higher than their designated share (some states have expanded food supplement programmes). Allocations for specific interventions are often spread across many budget heads, schemes, and ministries.	Inability to track investments across interventions and levels of government makes it difficult to know progress or benchmark requirements or spending.
**Budget execution**		
How much was received at each point in the public delivery chain?	This data is not in public domain or available in disaggregated form across levels and time periods.	The absence of data precludes benchmarking allocations data on fund releases and expenditures, and thereby isolating gaps. This affects the possibility of subsequent course correction as well as system accountability.
How much is spent?	Most expenditure is not in public domain. Data that is available is not disaggregated.	
**Budget evaluation**		
How well has the money been spent?	Data not publicly available nor usually disaggregated by components, levels and time periods.	Difficult to analyse how to improve system functioning and efficiency.
What bottlenecks exist in the system?	Information on bottlenecks varies across every block and district in the country. Data not available nor collected in any comparable way.	

These gaps are also evident in the limited research available on nutrition financing in India. While attempts have been made at costing analysis, these have had to rely on several assumptions in the absence of government data.^11 12^ Similarly, most budget analyses or PETs in India have been confined to smaller geographies or specific schemes.^35 36^ Thus, until the date of publication, there is no comprehensive nutrition budget analysis for all states across India.

## Conclusion

The exercise to map data availability with different phases of the PFM cycle found several gaps linked to a large degree with weaknesses in PFM. The absence of disaggregated data to track nutrition investments will have important implications on India’s commitment to reduce the incidence of malnutrition. While systemic reform of PFM systems is needed, these are likely to take some time. In the interim, the following steps can help ensure better data availability and thereby enable tracking progress on nutrition service delivery.

First, the best way to accurately estimate needs is through decentralised planning at the village level. This can be supported by building capacity and creating and enforcing accountability mechanisms such as social audits.

Second, there is a need for the government to expand data collection and improve data quality using existing platforms such as the HMIS, Rural Health Statistics (Annual statistics published by MoHFW covering various health related indicators, especially related to infrastructure and human resources). These platforms can be updated to track missing information, such as time spent by frontline workers on various tasks. These can feed into one comprehensive, robust PFM system in the public domain.

Third, using existing research better can help ensure comprehensiveness. Research on delivering public goods in low-resource contexts is ample, yet largely absent from current government documents. This includes studies that have provided estimates for missing costs, or provided evidence on public finance issues such as delays in fund releases, or access and distribution issues. Using such research on inefficiencies in implementation can help improve the tracking of nutrition finances.

Fourth, the success of nutrition interventions in India hinges on the transparency and public access to fiscal information. In Guatemala and Peru, transparent and regular publication of financial data and its accessibility spurred accountability across all levels and improved actual spending.^37^ Both researchers and governments should work towards making more data publicly available and ensure smoother feedback loops and collaborative efforts across states, local governments, and civil society organisations.

Lastly, some gaps in data can be overcome by the careful use of assumptions. These, however, must be deployed with caution as they are incomplete substitutes for actual figures. Relatedly, since data such as costs can vary significantly based on changing assumptions for prices, intervention components, scheme design and target populations, highlighting this variability is useful.

To conclude, we emphasise that mapping data availability across countries and contexts can improve the quality of research and subsequent policy-making, both of which are essential to reduce malnutrition.

## Data Availability

All data relevant to the study are included in the article.
